# A reactivity-selectivity study of the Friedel-Crafts acetylation of 3,3′-dimethylbiphenyl and the oxidation of the acetyl derivatives

**DOI:** 10.1186/1752-153X-6-52

**Published:** 2012-06-08

**Authors:** Salam JJ Titinchi, Fadhil S Kamounah, Hanna S Abbo, Ole Hammerich

**Affiliations:** 1 Department of Chemistry, University of the Western Cape, Private Bag X17, Bellville, 7535, South Africa; 2 CISMI, Department of Science, Systems and Models, Roskilde University, Universitetsvej 1, P.O. Box 260, Roskilde, DK-4000, Denmark; 3 Department of Chemistry, University of Copenhagen, Universitetsparken 5, Copenhagen Ø, DK-2100, Denmark

**Keywords:** Friedel-Crafts reaction, 3,3′-dimethylbiphenyl, Monoacetylation, Diacetylation, Carboxylic acids, DFT calculations

## Abstract

**Background:**

Friedel-Crafts acetylation is an important route to aromatic ketones, in research laboratories and in industry. The acetyl derivatives of 3,3′-dimethylbiphenyl (3,3′-dmbp) have applications in the field of liquid crystals and polymers and may be oxidized to the dicarboxylic acids and derivatives that are of interest in cancer treatment.

**Findings:**

The effect of solvent and temperature on the selectivity of monoacetylation of 3,3’-dmbp by the Perrier addition procedure was studied using stoichiometric amounts of reagents. 4-Ac-3,3′-dmbp was formed almost quantitatively in boiling 1,2-dichloroethane and this is almost twice the yield hitherto reported. Using instead a molar ratio of substrate:AcCl:AlCl_3_ equal to 1:4:4 or 1:6:6 in boiling 1,2-dichloroethane, acetylation afforded 4,4′- and 4,6′-diacetyl-3,3′-dmbp in a total yield close to 100%. The acetyl derivatives were subsequently converted to the carboxylic acids by hypochlorite oxidation. The relative stabilities of the isomeric products and the corresponding σ-complexes were studied by DFT calculations and the data indicated that mono- and diacetylation followed different mechanisms.

**Conclusions:**

Friedel-Crafts acetylation of 3,3′-dmbp using the Perrier addition procedure in boiling 1,2-dichloroethane was found to be superior to other recipes. The discrimination against the 6-acetyl derivative during monoacetylation seems to reflect a mechanism including an AcCl:AlCl_3_ complex or larger agglomerates as the electrophile, whereas the less selective diacetylations of the deactivated 4-Ac-3,3′-dmbp are suggested to include the acetyl cation as the electrophile. The DFT data also showed that complexation of intermediates and products with AlCl_3_ does not seem to be important in determining the mechanism.

## Findings

The Friedel-Crafts acylation is a powerful and successful way to introduce new carbon-carbon bonds in aromatic compounds and is one of the most important one-step routes for the synthesis of aromatic ketones, in research laboratories and in the chemical and pharmaceutical industries [1-5]. Although the reaction has been known for more than 130 years it still receives attention [6-16] the recent interest being focused on the use of ionic liquids as solvents [7], selectivity studies [6,9,11,13], the application of solid catalysts [16], and still, mechanism details [8,12,14,15]. Simple aromatic hydrocarbons and methyl substituted derivatives have featured as substrates in both earlier and more recent studies of the reaction including benzenes [6,7,17-19], biphenyls and fluorene [11,13,20], naphthalenes [21-23], anthracenes [14,24,25], phenanthrenes [12,26], pyrenes [27], and chrysenes [28] and as a part of our continuing interest in Friedel-Crafts acetylations [11,13,21,28-31], we now report details of the acetylation of 3,3′-dimethylbiphenyl (3,3′-dmbp). Compounds and materials derived from this building block are of particular interest in the field of molecular liquid crystals [31], polymers [32,33] and metal-complexes [34].

The literature offers only few studies of the Friedel-Crafts functionalization of 3,3′-dmbp. In the early thirties it was reported [35] that acylation of 3,3′-dmbp with α-naphthoyl chloride in the presence of AlCl_3_ resulted in the formation of 4,4′-di(1-naphthoyl)-3,3′- dmbp (41%) and to the best of our knowledge only two studies of the Friedel-Crafts acetylation of 3,3′-dmbp have appeared. Acetylation in 1,2-dichloroethane with acetic anhydride and aluminium chloride was reported [36] to give the 4-acetyl derivative (50%) and the same product was obtained (38.5%) when the reaction was carried out in carbon disulphide [37]. Diacetylation of 3,3′-dmbp has been mentioned only briefly in the literature [30]. Other electrophilic substitution reactions of 3,3′-dmbp include chlorination that gives a mixture of 2-, 4- and 6-chloro-3,3′-dmbp with the 4-isomer being dominating [38], bromination and iodination that lead to the 4-halo derivatives [39] and sulphonation [40] that with an excess of 98% H_2_SO_4_ leads to a 52:48 mixture of the 4,4′-and 4,6′-disulphonic acids.

Below we report the results of an investigation of the distribution of the isomers formed by Friedel-Crafts mono- and diacetylation of 3,3′-dmbp using the Perrier addition procedure, that is the addition of the hydrocarbon to the preformed acetyl chloride-aluminium chloride complex. The study included the effect of solvent and temperature on yields and product distribution. The selectivity of the mono- and diacetylation processes is discussed using theoretical DFT data for the relative stability of the isomeric products and the intermediate σ-complexes. The further oxidation of the resulting mono- and diketones with hypochlorite to the corresponding carboxylic acids is reported as well.

### Synthesis of 3,3′-dimethylbiphenyl

The starting material, 3,3′-dmbp, was synthesized by two different methods. Ullman coupling of 3-iodotoluene yielded 60% of a high purity product (99.5% glc), while deamination of 4,4′-diamino-3,3′-dmbp resulted in a somewhat higher yield, 84%. However the latter procedure is slightly more expensive due to the high cost of starting materials.

### The acetylation products

**Scheme 1:**
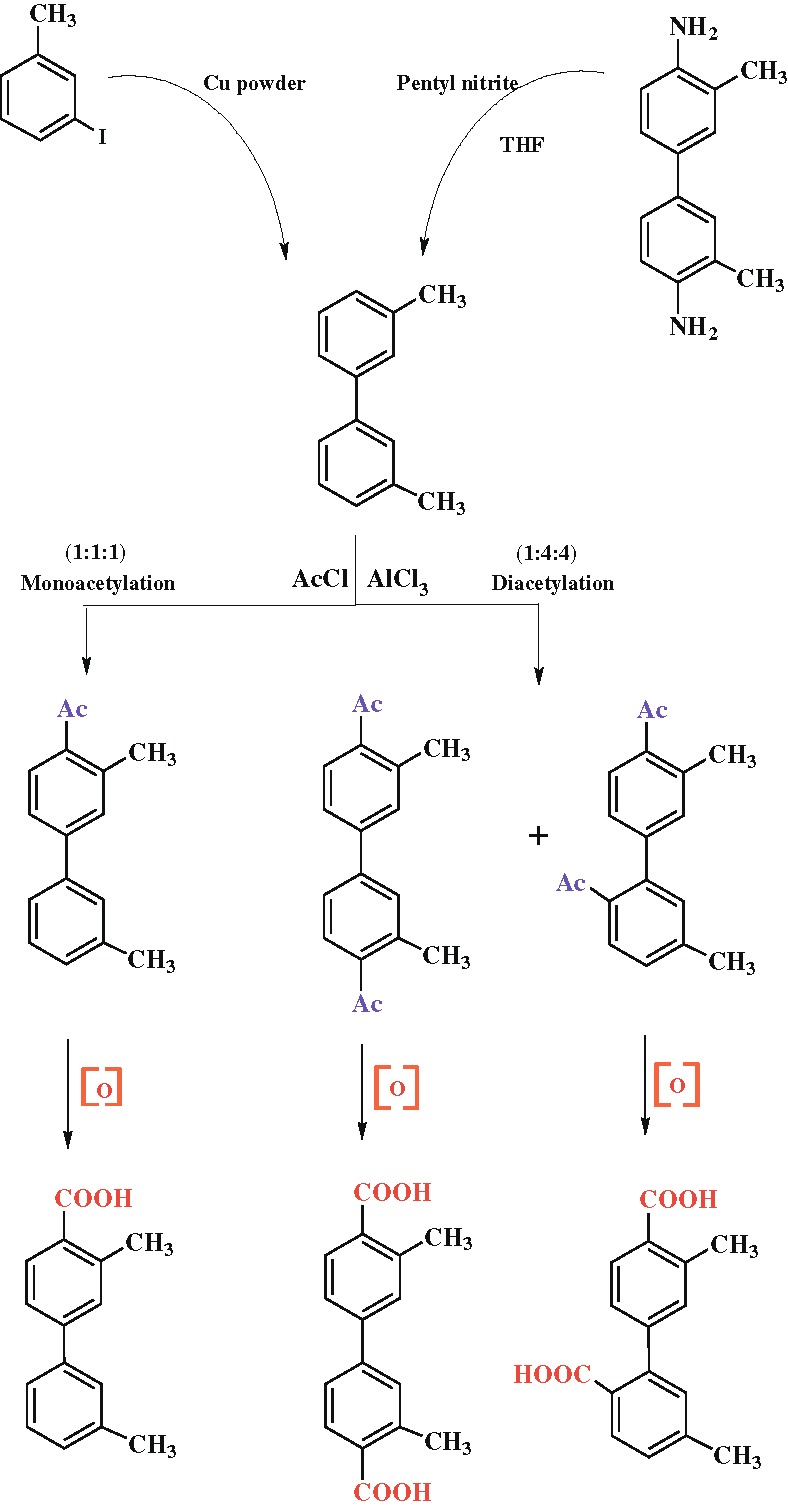
Synthesis route of the ketones and carboxylic acids.

The products and yields are summarized in Tables 1 and 2. The abbreviations used for the mono and diketones are x-Ac and x,y′-diAc, respectively, where x and y′ refer to the acetylated positions of the two phenyl rings. The route for the synthesis of the acetyl isomer and the corresponding carboxylic acids is depicted in Scheme 1.

**Table 1:** **Results from Friedel-Crafts acetylation of 3,3′-dimethylbiphenyl using the Perrier addition procedure**^**a**^

**Run No**	**Solvent**	**Temp (°C)**	**Yield (%)**	**Isomer distribution (%) 4-Ac**
1	ClCH_2_CH_2_Cl	25	56.5	100
2	CS_2_	25	55.1	100
3	CH_3_NO_2_	25	57.5	100
4	ClCH_2_CH_2_Cl	45	51.9	100
5	CS_2_	45	60.8	100
6	CH_3_NO_2_	45	47.2	100

^a^ The 3,3′-dmbp:AcCl:AlCl_3_ molar ratio was 1:1:1 and the reaction time was 3 h.

**Table 2:** **Results from Friedel-Crafts acetylation of 3,3′-dimethylbiphenyl in the boiling reaction mixture using the Perrier addition procedure**^**a**^

**Run No**	**Solvent**	**Bp**^**b**^**(°C)**	**Yield (%)**	**Isomer distribution (%)**		
				**4-Ac**	**4,4′-diAc**	**4,6′-diAc**
7	CH_2_Cl_2_	40	68.9	100	----	----
8	ClCH_2_CH_2_Cl	84	96.5	100	----	----
9	Cl_2_CHCHCl_2_	147	68.2	100	----	----
10	C_6_H_5_Cl^c^	131	58.6	100	----	----
11	CS_2_	46	55.4	100	----	----
12	CH_3_NO_2_^d^	101	11.8	100	----	----
13	C_6_H_5_NO_2_^d^	211	Trace	Trace	----	----
14	ClCH_2_CH_2_Cl^e^	84	99.9	1.6	43.3	55.1
15	ClCH_2_CH_2_Cl^f^	84	92.4	----	37.6	62.4

^a^ The 3,3′-dmbp:AcCl:AlCl_3_ molar ratio was 1:1:1 unless otherwise stated and the reaction time was 23 h. ^b^ Boiling point of the neat solvent. ^c^ In addition 4-chloroacetophenone was formed in ~20% yield. ^d^ The major products were dark colored polymeric materials and other unidentified compounds. ^e^ The 3,3′-dmbp:AcCl:AlCl_3_ molar ratio was 1:4:4. ^f^The 3,3′-dmbp:AcCl:AlCl_3_ molar ratio was 1:6:6.

As seen from Table 1 acetylation of 3,3′-dmbp at 25°C and 45°C using equimolar amounts of 3,3′-dmbp, AcCl and AlCl_3_ in 1,2-dichloroethane, carbon disulfide and nitromethane gave the 4-acetyl isomer, 4-Ac, exclusively in yields in the 50-60% range. At higher temperature, that is at the boiling point of the reaction mixture, 4-Ac was still the only ketone isolated after work-up (Table 2) and in 1,2-dichloroethane the yield had now increased to almost 100% (run no 8). This is a considerable improvement over the yield reported earlier (50%) resulting from acetylation at 50^o^ C with acetic anhydride in the same solvent [36]. In chlorobenzene, the 4-Ac (58.6%) was accompanied by the formation of 4-chloroacetophenone as a byproduct in about 18% yield formed by acetylation of the solvent. In boiling nitromethane the yield was only 11.8% and the reaction was accompanied by the formation of a dark polymeric material. The low yield obtained in nitromethane is not unusual; it was observed also in previous studies of the acetylation of 4,4′-dimethylbiphenyl [11] and of 9H-fluorene [13]. When the reaction was carried out in boiling nitrobenzene acetylated products could only be detected in trace amounts. Probably the high temperature (bp. 211°C) in this latter case was causing degradation of substrate, intermediates or products.

When acetylation was carried out in boiling 1,2-dichloroethane with an excess of AcCl and AlCl_3_ (the molar ratio 3,3′-dmbp:AcCl:AlCl_3_ equal to 1:4:4 or 1:6:6), and conditions that are otherwise the same, a mixture of 4,4′- and 4,6′-diAc in a total yield close to 100% was obtained with the relative yield of the 4,6′-isomer increasing slightly with increasing amounts of the AcCl:AlCl_3_ mixture (run no 14 and 15).

Thus, similarly to what has been observed earlier [36,37] we find that acetylation of 3,3′-dmbp takes place in the 4-position and the substitution pattern we observe for diacetylation, 4,4′-and 4,6′-, is the same as that reported earlier for sulfonation [40].

### The spectral data

The ^1^ H NMR spectral data of the acetyl derivatives illustrate that the chemical shift of the methyl protons could be used as an indication of the position of substitution of an acetyl or a methyl group linked to the aromatic rings [30,41,42]. Analysis of the ^1^ H NMR data showed that the methyl protons chemical shifts (δCH_3_) for all the three isomers are shifted downfield by 0.15 ppm compared to the methyl protons in the parent compound. This confirms that the acetyl group is situated ortho to the methyl group, whereas the δCH_3_ for the second ring in 4,6′-diAc-3,3′-dmbp remains almost unaffected (0.49 ppm) as it is far from the methyl group. The results obtained from the ^1^ H NMR are in agreement with the methyl protons chemical shifts (δCH_3_) in toluene viz. the δCH_3_ being shifted downfield from 2.34 to 2.85 ppm on substitution of an acetyl group ortho to the methyl group, while for meta- and para-substitution, the values are 2.27 and 2.52 ppm, respectively [43].

The IR spectroscopy data further supports the positional assignment. An intense absorption band of the C = O stretching vibration of the acetyl group appeared at 1685–1650 cm^−1^, which elaborate a carbonyl group sterically hindered by the adjacent tolyl or methyl groups. The carbonyl band shifted to a lower wave number in comparison to acetophenone (1690 cm^−1^) [44]. In fact, the assignment is in good agreement with the expectation of the orientation during the acetylation reaction due to the directing effect of the methyl and tolyl substituents.

The mass spectra also support the structural assignment by existence of the molecular ion peak [M]^+^. The breakdown of the two monoacetyl isomers under electron impact follows almost the same mass fragmentation pathways. The major fragment ions observed in the mass spectra were [M-CH_3_]^+^, [M-COCH_3_]^+^, [M-(CH_3_ + COCH_3_)]^+^, [PhCH_3_]^+^, [C_6_H_4_]^+^ and [COCH_3_]^+^.

### The relative stability of the products

It is well known that the ketones resulting from Friedel-Crafts acetylations exist in the reaction mixtures as the AlCl_3_ complexes [5,7,25,45] and for that reason the discussion below of the relative stability of the products includes not only the mono- and diketones, x-Ac and x,y′-diAc, as isolated after work-up, but also the AlCl_3_ complexes; the 1:1 AlCl_3_ complexes for the monoketones and the 1:1 and 1:2 complexes for the diketones.

Theoretical data obtained by DFT B3LYP 6-31 G(d,p) calculations for the mono- and diketones are summarized in Tables 3 and 4; the results for the AlCl_3_ complexes are included in Additional file 1: Scheme S1, Tables S1, S2 and S3.

**Table 3:** Total energies, Gibbs free energies (298 K), and structural properties for 3,3′-dimethylbiphenyl and the monoacetyl isomers, x-Ac

**Substituent**	**Total energy E (a.u.)**	**G**_**298**_**(a.u.)**	**G**_**298**_**relative to 5-Ac (kJ mol**^**−1**^**)**	**θ**^**a**^**(degrees)**	**ϕ**^**b**^**(degrees)**
None	−541.963044	−541.768297	−	38.7	−
2-Ac	−694.601204	−694.371990	33.7	49.1	60.8
4-Ac	−694.610042	−694.380270	12.0	37.3	0.2
5-Ac	−694.612983	−694.384835	0	39.0	0.7
6-Ac	−694.604265	−694.376276	22.5	54.4	31.3

Results from DFT B3LYP 6-31 G(d,p) calculations. ^a^Dihedral angle between the two benzene rings taken as the average of the C2-C1-C1′-C6′ and C6-C1-C1′-C2′ dihedral angles. ^b^Dihedral angle between the carbonyl group and the benzene ring to which it is attached taken as the C_Ar_-C_Ar_-C = O dihedral angle.

**Table 4:** Total energies, Gibbs free energies (298 K), and structural properties for the acetyl substituted 4-acetyl-3,3′-dimethylbiphenyls, 4,y′-diAc

**Substituents**	**Total energy E (a.u.)**	**G**_**298**_**(a.u.)**	**G**_**298**_**relative to 4,5′- diAc (kJ mol**^**−1**^**)**	**θ**^**a**^**(degrees)**	**ϕ**^**b**^**(degrees)**	
					**4-Ac**	**y′-Ac**
4,2′-diAc	−847.247523	−846.984334	35.3	48.2	0.1	61.5
4,4′-diAc	−847.256356	−846.992372	14.2	36.5	1.0	1.0
4,5′-diAc	−847.259523	−846.997775	0	36.7	0.7	0.4
4,6′-diAc	−847.250427	−846.988520	24.3	53.0	0.6	32.4

Results from DFT B3LYP 6-31 G(d,p) calculations. ^a^Dihedral angle between the two benzene rings taken as the average of the C2-C1-C1′-C6′ and C6-C1-C1′-C2′ dihedral angles. ^b^Dihedral angle between the carbonyl group and the benzene ring to which it is attached taken as the C_Ar_-C_Ar_-C = O dihedral angle.

The data in Table 3 show that the biphenyl dihedral angles, θ, for 4-Ac and 5-Ac are close to that for unsubstituted 3,3′-dmbp. All fall in the range 37–39^o^, which is within the range of values determined for 3,3′-dmbp by photoelectron [46-48] or NMR spectroscopy [49,50]. It is seen also that the C_Ar_-C_Ar_-C = O dihedral angle, ϕ, between the carbonyl group and the benzene ring to which it is attached is close to 0^o^ for 4-Ac and 5-Ac showing that the steric interactions between the 4-Ac group and the neighboring hydrogen and methyl, and between the 5-Ac group and the two neighboring hydrogens, are negligible. In contrast, the geometries for the two other isomers, 2-Ac and 6-Ac, are both affected by steric interactions between the acetyl group and the 2′- and 6′-hydrogens in the neighboring ring. These interactions cause θ to increase from approximately 38^o^ to 49^o^ (2-Ac) and 54^o^ (6-Ac) and steric interactions also hinder co-planarity of the carbonyl group and the aromatic ring as reflected by ϕ values as high as 60.8^o^ (2-Ac) and 31.3^o^ (6-Ac). As a consequence of this both 2-Ac and 6-Ac suffer from diminished electronic conjugation resulting in values of G_298_ that are 33.7 and 22.5 kJ mol^−1^, respectively, higher than G_298_ for the lowest energy isomer, 5-Ac. It is seen also that 5-Ac is 12 kJ mol^−1^ more stable than 4-Ac, the only isomer isolated after work-up. The stability of the four monoacetyl derivatives decreases in the order 5 > 4 > 6 > 2 and thus, the isomer with an 1,3,5-arrangement of the three substituents (Ac, Me and Ar) in the acetylated benzene ring is found to be the most stable reminiscent of the relative stability of trialkylbenzenes [51].

Complexation with AlCl_3_ has only a minor effect on the structure of the monoketones (Additional file 1: Table S1), but we do notice that 2-AcAlCl_3_ and 4-AcAlCl_3_ are both destabilized relative to the two other AlCl_3_ complexes presumably owing to steric interactions between the bulky AlCl_3_ group and the neighboring 3-CH_3_ group. For 4-AcAlCl_3_ these interactions also cause ϕ to increase from ~0 to 11.4^o^. However, the order of decreasing stability of the AlCl_3_ complexes remains the same as that for the uncomplexed ketones.

Comparison of the data in Table 3 for the monoketones with those in Table 4 for the diketones shows that the presence of the 4-Ac group has only little effect on the geometry, and the relative stability of the diketones is found to decrease in the same order as before, that is 4,5′ > 4,4′ > 4,6′ > 4,2′. Similarly, by comparison of the data in Additional file 1: Table S1 for the AlCl_3_ complexes of the monoketones with those in Tables S2 and S3 for the AlCl_3_ complexes of the diketones we see no new effects on the structure and the relative stability caused by complexation of the diketones with AlCl_3_.

Thus, it is clear that the Friedel-Crafts mono- and diacetylation of 3,3′-dmbp with AcCl and AlCl_3_ under the Perrier conditions does not favor the formation of the most stable ketones or ketone AlCl_3_ complexes and thus appears, as expected, to be under kinetic control.

### The relative stability of the σ-complexes

**Scheme 2:**
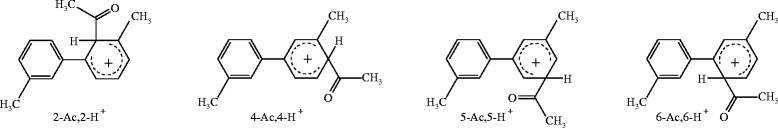
The σ-complexes for monoacetylation.

Let us now briefly examine the classical ionic mechanism for acetylation and test whether the distribution of the products is indeed reflected by the relative stability of the σ-complexes shown in Scheme 2 (for mono-substitution only) as one might expect for a kinetically controlled reaction. The relative stabilities of the four 

The relative stabilities of the four σ-complexes leading to monoacetylation are summarized in Table 5 (the data for the corresponding AlCl_3_ complexes are included Additional file 1: Scheme S2 and Table S4). The order of decreasing stability is seen from Table 5 to be 4 ≈ 6 > 2 > 5 in agreement with the observation that 5-Ac is not formed in spite of the fact that this is the most stable isomer as already mentioned above, but it is noticed also that G_298_ for 4-Ac,4-H^+^ that leads to the only product formed is only 0.4 kJ mol^−1^ lower than G_298_ for 6-Ac,6-H^+^. The same is true for the AlCl_3_ complexes, x-AcAlCl_3_,x-H^+^ (Additional file 1: Table S4). Thus, the theoretical data for the relative stability of the σ-complexes for monoacetylation would predict the formation of both 4-Ac and 6-Ac as major products. This is in contrast to the experimental observation that 4-Ac is formed exclusively and therefore it appears that a feature of the reaction, so far not accounted for, causes the discrimination against the formation of 6-Ac. Here we wish to emphasize that the small energy differences found for 4-Ac,4-H^+^ and 6-Ac,6-H^+^, and for 4-AcAlCl_3_,4-H^+^ and 6-AcAlCl_3_,6-H^+^, do not appear to be a computational artifact. More advanced computational strategies including the application of the larger 6-311 + G(d,p) basis set or the inclusion of AlCl_4_^−^ ion pairs and PCM solvation result in energy differences that are still very small. Thus, the similarity of the energies of the two cations seems to be real. We will return to the implications of this result below.

**Table 5:** **Total energies, Gibbs free energies (298 K), and structural properties for the σ-complexes, x-Ac,x-H**^**+**^

**Substituent**	**Total energy E (a.u.)**	**G**_**298**_**(a.u.)**	**G**_**298**_**relative to 4- Ac,4-H**^**+**^**(kJ mol**^**−1**^**)**	**θ**^**a**^**(degrees)**
2-Ac,2-H^+^	−694.942784	−694.702831	7.3	24.9
4-Ac,4-H^+^	−694.945838	−694.705627	0	23.3
5-Ac,5-H^+^	−694.929221	−694.692290	35.0	36.8
6-Ac,6-H^+^	−694.944629	−694.705485	0.4	32.3

Results from DFT B3LYP 6-31 G(d,p) calculations. ^a^Dihedral angle between the two benzene rings taken as the average of the C2-C1-C1′-C6′ and C6-C1-C1′-C2′ dihedral angles.

The theoretical data for the four σ-complexes leading to diacetylation are summarized in Table 6 (the data for the corresponding AlCl_3_ complexes are included Additional file 1: Scheme S2, Tables S5 and S6).

**Table 6:** **Total energies, Gibbs free energies (298 K), and structural properties for the σ-complexes, 4-Ac,y′-Ac,y′-H**^**+**^

**Substituent**	**Total energy E (a.u.)**	**G**_**298**_**(a.u.)**	**G**_**298**_**relative to 4-Ac,4′- Ac,4′-H**^**+**^**(kJ mol**^**−1**^**)**	**θ**^**a**^**(degrees)**
4-Ac,2′-Ac,2′-H^+^	−847.582972	−847.310553	6.6	31.8
4-Ac,4′-Ac,4′-H^+^	−847.585556	−847.313061	0	23.9
4-Ac,5′-Ac,5′-H^+^	−847.571021	−847.300305	33.5	36.5
4-Ac,6′-Ac,6′-H^+^	−847.584905	−847.312773	0.8	32.3

Results from DFT B3LYP 6-31 G(d,p) calculations. ^a^Dihedral angle between the two benzene rings taken as the average of the C2-C1-C1′-C6′ and C6-C1-C1′-C2′ dihedral angles.

Comparison of the data in Tables 5 and 6 shows that the presence of the 4-Ac group, as also intuitively expected, does not have a significant effect on the relative stability of the σ-complexes that decreases in the same order as for monoacetylation, that is 4,4′ ≈ 4,6′ > 4,2′ > 4,5′. The same is true for the AlCl_3_ complexes, but we do notice (Additional file 1: Table S5) that the 1:1 complexes that involve the acetyl group resulting from the electrophilic attack are of significantly higher energies than those involving the 4-Ac group and therefore appear to be of only minor importance. Thus, in contrast to the monoacetylation the theoretical data for diacetylation are indeed in agreement with the assumption that the product distribution, that is the formation 4,4′-diAc and 4,6′-diAc in similar amounts, reflects the relative stability of the σ-complexes. In other words, the classical ionic mechanism may well be in operation for the diacetylation process.

**Scheme 3:**
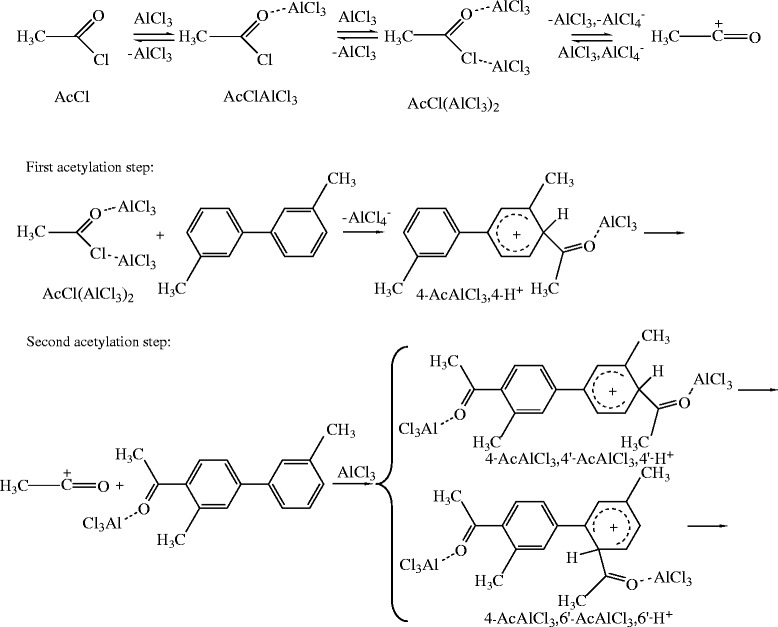
The initial steps of the mechanisms suggested for the first and second acetylation of 3,3′-dmbp.

The problem that remains is the effective discrimination against 6-Ac during monoacetylation. Here we should bring into mind that the composition of the AcCl-AlCl_3_ mixtures, such as those used under the Perrier conditions, may contain not only the free acetyl cations, but also ion-pairs involving this species as well as acetylating species resulting from the complexation between AcCl and AlCl_3_ and possibly even larger agglomerates, the actual distribution of these species of course being dependent on the solvent and the concentration ratio of AcCl and AlCl_3_. We find it likely that acetylation of the unsubstituted 3,3′-dmbp may involve such larger acetylation species reminiscent of the substitution mechanism [5] and that the discrimination against attack at the 6-position is caused by steric hindrance, whereas the deactivated 4-Ac derivative requires the smaller, more potent, and thus less discriminating, acetyl cation as in the ionic mechanism. This is illustrated in Scheme 3. We are presently engaged in more detailed experimental and theoretical studies of this problem.

## Experimental

### Materials

4,4′-Diamino-3,3′-dimethylbiphenyl, 3-methylaniline, pentyl nitrite, acetyl chloride, anhydrous aluminium chloride, copper powder, potassium hydroxide, sodium hypochlorite (6% available chlorine), silica gel and THF were from BDH and chloroform, dichloroethane, hydrochloric acid, benzene and petroleum ether were from Aldrich. Silica gel used for column chromatography was 230–400 mesh ASTM from Merck. All solvents used were of analytical purity from Fluka and were dried over anhydrous calcium chloride or anhydrous sodium sulfate prior to use in the acetylation reactions.

### Instrumentation

IR spectra were measured as KBr discs or as thin films of Nujol on a Pye Unicam sp3-300 spectrophotometer. ^1^ H NMR spectra were recorded on a Varian FT-80 MHz and Bruker 100 MHz for solutions in deuterated chloroform, using tetramethylsilane as an internal standard. GLC analyses were carried out with a stainless steel column (2 m × 2.2 mm i.d.) packed with SE-30 (10%) on acid-washed Chromosorb W (80–100 mesh). Nitrogen (15 lb in^−2^) was used as carrier gas at 250°C; a Pye Unicam 204 instrument fitted with flame ionization detector was used. Peak areas were measured by Pye Unicam DP 88 electronic integrator. Mass response towards the different compounds was determined and appropriate corrections were applied. Elemental analyses were carried out in Alfred Bernhard Mikroanalytisches Laboratorium, Germany. Mass spectra were obtained on a VG MassLab 12–250 GC mass spectrometer.

### General acetylation procedure

The route for the synthesis of the ketones and carboxylic acids is depicted in Scheme 1.

The Friedel-Crafts acetylations were carried out in a three-necked round-bottomed flask placed in oil bath and fitted with a dropping funnel, a thermometer and a reflux condenser with a calcium chloride absorption trap. Equimolar quantities of the reactants in the dry solvent (20 mL) were brought together under the Perrier conditions, where the catalyst and acylating agent were allowed to react prior to addition of the substrate. A stoichiometric amount of 3,3′-dmbp in the same solvent (20 mL) was added drop wise over a period of 5 min to the stirred reaction mixture at the desired temperature. Stirring was continued at the same temperature for the total time shown in the Tables below. The mixture was then added to an excess of crushed ice and 3 M HCl. The organic phase was separated and the water phase was washed with the organic solvent. The combined organic phases were then washed with water (5 × 50 mL), dried with anhydrous sodium sulfate and finally the solvent was removed at reduced pressure using a rotary evaporator. When nitromethane was used as the solvent, the organic layer was washed with (2 × 100 mL) of 3 M NaOH instead of water. The viscous residue was dissolved in benzene and passed through a short column of silica gel to remove any polymeric materials. The reaction mixtures were examined for the content of ketones by GLC analysis. The symmetry of 3,3′-dmbp molecule limits the number of isomeric monosubstituted derivatives to four at 2-, 4-, 5-and 6- position and the identity of each component was established by comparison of the retention time with that of authentic samples and by oxidizing the ketones to the corresponding carboxylic acids. The overall yield of products is presented in Tables 1 and 2 above . The mono-and diketones synthesized were identified by their ^1^ H NMR, IR, mass spectra and by elemental analysis.

### Preparations

#### Preparation of 3,3′-dimethylbiphenyl

Two different methods, A and B below, for the preparation of 3,3′-dmbp was followed, both of which gave identical products. (A): 3,3′-dmbp was prepared by deamination of 4,4′-diamino-3,3′-dmbp following a published procedure [52]. 4,4′-diamino-3,3′-dmbp (4.24 g, 0.02 mol) in THF (50 mL) was added drop-wise within 2 h to a boiling solution of pentyl nitrite (23.4 g, 0.2 mol) in THF (30 mL). The obtained mixture was refluxed for 6 h. Distillation of the solvent resulted in a viscous dark brown liquid. The viscous liquid was extracted with chloroform then the solvent was evaporated. The remaining material was distilled under vacuum. An oily colorless liquid of 3,3′-dmbp was obtained (3.1 g, 84%). (B): Ullmann synthesis starting from 3-methylaniline. 3-iodotoluene was prepared from 3-methylaniline using the general method [36] where a colorless liquid was obtained; bp. 214°C (lit. [53] 213°C). A mixture of 3-iodotoluene (42 g, 0.2 mol) and copper powder (24.4 g, 0.4 mol) was refluxed for 72 h. After cooling the mixture was dissolved in chloroform and filtered off. On evaporation of the solvent a brown liquid was obtained distilled under vacuum which gave a colorless liquid of 3,3′-dmbp (21 g, 60%); b.p. 146°C/17 mmHg. (lit. [54] 135°C/3 mmHg) (Found C, 92.16; H, 7.71%; C_14_H_14_ requires C, 92.26; H, 7.74%). δ (CDCl_3_) 2.43 (6 H, s, 3-CH_3_ and 3′-CH_3_), 7.17-7.31 (6 H, m, aromatic H), 7.53 (d, J = 7.8 Hz, 2 H, H-6,6′).

#### 4-Acetyl-3,3′-dimethylbiphenyl

To a stirred solution of acetyl chloride (0.392 g; 0.005 mol) and aluminium chloride (0.666 g; 0.005 mol) in 1,2-dichloroethane (20 mL), 3,3′-dmbp (0.91 g; 0.005 mol) in the same solvent (20 mL) was added and the mixture was stirred at room temperature for 1 h and then refluxed for 22 h. The mixture was then added to an excess of crushed ice and 3 M HCl. The organic phase was separated, the extract was added to the washing (solvent) of the acid layer and the combined extracts were washed with water (5 × 50 mL) and the solvent removed by rotary evaporator. The dark brown oil obtained was dissolved in benzene, chromatographed over silica gel/petroleum ether first to get rid of the remaining starting hydrocarbon, and then the ketone was obtained by using benzene as an eluent. Evaporation of benzene affords pure 4-acetyl-3,3′-dmbp (0.98 g; 87.1%) as a yellow oil: Found C, 85.54; H, 7.19%; C_16_H_16_O requires C, 85.68; H, 7.19%). ν_max_ (neat) 1680 (C = O) cm^−1^; δ (CDCl_3_) 2.43 (s, 3 H, 3′-CH_3_), 2.58 (s, 6 H, 3-CH_3_ and 4-CH_3_CO), 7.17–7.53 (6 H, m, aromatic H), 7.78 (d, J_5,6_ = 9 Hz, 1 H, H-6′). m/z 224 [M]^+^, 209 [base peak, M-CH_3_]^+^, 181 [M − COCH_3_]^+^, 151 [M-(2CH_3_ + COCH_3_)]^+^, 89 [PhCH_3_]^+^, 76 [C_6_H_4_]^+^, 43 [COCH_3_]^+^.

#### 3,3′-Dimethylbiphenyl-4-carboxylic acid

A mixture of 4-acetyl-3,3′-dmbp (2.1 g; 0.009 mol), potassium hydroxide (1.5 g) and sodium hypochlorite (150 mL) was heated for 3 h. Additional quantities of hypochlorite solution (45 mL) were added to the mixture after 1 and 2 h. After cooling, the solution was acidified with 50% HCl and the precipitate was extracted with ether (4 × 25 mL), washed with water then with dilute potassium hydroxide. The basic layer was separated, acidified with dilute HCl and a precipitate was formed which was extracted with ether. The acid was obtained after ether evaporation as yellow crystals (1.13 g; 53%), mp 168–170°C. ν_max_ (KBr) 1683 (C = O) and 3040 (OH) cm^−1^ δ (CDCl_3_) 2.45 (s, 3 H, 3′-CH_3_), 2.73 (s, 3 H, 3-CH_3_), 7.25–7.47 (m, 6 H, aromatic H), 8.14 (d, J_5_′_,6_′ = 8.5 Hz, 1 H, H-6′),11.69 (s, 1 H, 4-COOH exchangeable with D_2_O). (Found C, 79.62; H, 6.24%; C_15_H_14_O_2_ requires C, 79.65; H, 6.19%).

#### 4,4′- and 4,6′-diacetyl-3,3′-dimethylbiphenyl

To a stirred solution of acetyl chloride (3.136 g; 0.04 mol) and aluminium chloride anhydrous (5.323 g; 0.04 mol) in 1,2-dichloroethane (40 mL), 3,3′-dmbp (1.82 g; 0.01 mol) in the same solvent (40 mL) was added drop wise over 5 minutes. The mixture was stirred at room temperature for 1 h then gently refluxed for 22 h. At the end of the reaction time, the resulting mixture was cooled and added to a mixture of conc. HCl and ice. The dark brown viscous oil obtained was chromatographed over silica gel/benzene. The yellow oil obtained on evaporation of benzene. A yellow solid (0.86 g, m.p. 129–131°C) was obtained on adding a little of benzene. Recrystallization of the yellow solid using ether gave 4,4′-diacetyl-3,3′-dmbp as yellow needles (0.79 g, 29%) m.p. 131–3°C. (lit. [55] 136°C). Found C, 81.11; H, 6.74%; C_18_H_18_O_2_ requires C, 81.17; H, 6.81%). ν_max_ (KBr) 1684 (C = O) cm^−1^ δ (CDCl_3_) 2.62 (s, 12 H, 3-CH_3_, 3′-CH_3_, 4-COCH_3_, 4′-COCH_3_), 7.29–7.61 (m, 4 H, aromatic H), 7.83 (d, J_5,6_ and J_5_′_,6_′ = 8.9 Hz, 2 H, H-5, 5′). The mother liquor was subjected to column chromatography, which gave rise to, first, the 4,4′-diacetyl-3,3′-dmbp and, second, 4,6′-diacetyl-3,3′-dmbp (1.13 g, 42.5%). (found C, 81.03; H, 6.71%; C_18_H_18_O_2_ requires C, 81.17; H, 6.81%). ν_max_ (KBr) 1685 (C = O) cm^−1^ δ (CDCl3) 2.00 (s, 3 H, 3′-CH_3_), 2.39 (s, 3 H, 3-CH_3_), 2.56 (s, 6 H, 4-COCH_3_, 6′-COCH_3_), 7.14–7.29 (m, 4 H, aromatic H), 7.52 (d, J_4_′_,5_′ = 9 Hz, 1 H, H-5′), 7.71 (d, J_5,6_ = 9 Hz, 1 H, H-5). m/z 266 [M]^+^, 251 [base peak, M-CH_3_^+^, 209 [M-(CH_3_ + COCH_3_^+^, 164 [M-(2CH_3_ + COCH_3_)]^+^, 151 [M-(2CH_3_ + 2COCH_3_)]^+^, 118 [PhCOCH_3_^+^, 89 [PhCH_3_^+^, 76 [C_6_H_4_^+^, 43 [COCH_3_^+^.

#### 3,3′-Dimethylbiphenyl-4,4′-dicarboxylic acid

A mixture of 4,4′-diacetyl-3,3′-dmbp (0.2 g; 0.007 mol), potassium hydroxide (1.0 g) and sodium hypochlorite (100 mL) was heated for 4 h. Additional quantities of hypochlorite solution (30 mL) were added to the mixture after 1, 2 and 3 h. After cooling, the solution was acidified with 50% HCl and the precipitate was extracted with ether (4 × 25 mL), washed with water then with dilute potassium hydroxide. The basic layer was separated, acidified with dilute HCl and a white precipitate was formed and filtered. The solid acid obtained was recrystallized from benzene which gave a white crystals of 3,3′-dmbp-4,4′-dicarboxylic acid. (0.09 g; 44.3%), mp 190°C(d). ν_max_ (KBr) 1687 (C = O) and 3050 (OH) cm^−1^ (found C, 71.56; H, 5.28%; C_16_H_14_O_4_ requires C, 71.11; H, 5.19%). δ (CDCl_3_) 2.67 (s, 6 H, 3-CH_3_ and 3′-CH_3_), 7.25–7.67 (m, 4 H, aromatic H), 8.04 (d, J_5,6_ = 8.5 Hz, 2 H, H-5 and H-5′), 11.83 (s, 2 H, 4- and 4′-COOH exchangeable with D_2_O)

#### 3,3′-Dimethylbiphenyl-4,6′-dicarboxylic acid

A mixture of 4,6′-diacetyl-3,3′-dmbp (0.2 g; 0.007 mol), potassium hydroxide (1.0 g) and sodium hypochlorite (100 mL) was heated for 5 h. Additional quantities of hypochlorite solution (30 mL) were added to the mixture after 1, 2, 3 and 4 h. After cooling, the solution was acidified with 50% HCl and the precipitate was extracted with ether (4 × 25 mL), washed with water then with dilute potassium hydroxide. The basic layer was separated, acidified with dilute HCl and a white precipitate was formed and filtered. The white solid obtained was recrystallized from ether/benzene which gave a white crystals of 3,3′-dmbp-4,6′-dicarboxylic acid. (0.076 g; 37.3%), mp >340°C (d). ν_max_ (KBr) 1690 (C = O) and 3050 (OH) cm^−1^. Found C, 70.96; H, 5.30%; C_16_H_14_O_4_ requires C, 71.11; H, 5.19%). δ (CDCl_3_) 2.44 (s, 3 H, 3′-CH_3_), 2.62 (s, 3 H, 3-CH_3_), 7.15–7.70 (m, 5 H, aromatic H), 8.04 (d, J_5,6_ = 8.5 Hz, 1 H, H-5), 11.89 (s, 2 H, 4- and 6′-COOH exchangeable with D_2_O).

### DFT calculations

All calculations were carried out using the Gaussian 03 package [x86-Linux-G03RevB.05] of programs [56] installed on a PC cluster or the equivalent G03W suite of programs [version 6.1] installed on standard personal computers. The structure optimizations included the default or the gdiis procedures; geometrical constraints were not imposed. Thermochemical data at T = 298 K were obtained by frequency calculations. The conformational space for the compounds investigated is large and a number of local energy minima were detected. The conformational analysis in this study included the effects (i) the syn/anti orientation of the two methyl-substituted rings, (ii) the orientation of the carbonyl group relative to the plane of the aromatic ring to which it is attached and (iii) the orientation of the carbonyl group in the σ-complexes. Data are given only for the conformers that we have found to be of lowest energy. With respect to the orientation of the acetyl group relative to a neighboring methyl substituent we find that the conformation having the carbonyl oxygen atom pointing towards the methyl group is preferred in agreement with the results obtained by others [57].

## Conclusions

Friedel-Crafts acetylation of 3,3′-dmbp by the Perrier addition procedure in which a solution of the substrate was added to a stirred solution of the preformed complex of acetyl chloride and aluminium chloride has been investigated in detail. At 25, 45°C and higher temperature (reflux conditions) with stoichiometric amounts of reagents, acetylations give 4-acetyl-3,3′-dmbp entirely with almost quantitative yield obtained in boiling 1,2-dichloroethane. In 1,2-dichloroethane at reflux conditions with a hydrocarbon:acetyl chloride:aluminium chloride molar ratio of 1:4:4 or 1:6:6 acetylation affords 4,4′- and 4,6′-diacetyl-3,3′-dmbp as the sole products. The mono- and diketones were subsequently converted to the corresponding 3,3′-dmbp dicarboxylic acids by hypochlorite oxidation. Two of the compounds, 4,6′-diacetyl-3,3′-dmbp and the dicarboxylic acid derived from this species are reported for the first time. The relative stability of the possible acetylation products as evaluated by DFT B3LYP 6–31 G(d,p) calculations indicates that the acetylations do not favor the formation of the most stable products and are under kinetic control.

## Competing interests

The authors declare that they have no competing interests.

## Authors’ contributions

ST contributed to the findings and experimental part of the manuscript. FK contributed in the characterization of the compounds and contributed to the findings and experimental part of the manuscript. HA carried out the experimental work. OH carried out the computational work and contributed to the findings part the manuscript. All authors read and approved the final manuscript.

## Supplementary Material

Additional file 1 **Scheme S1.** Illustrations of the AlCl_3_ complexes of the ketones. **Table S1.** Total energies, Gibbs free energies (298 K) and structural properties for the AlCl_3_ complexes, x-AcAlCl_3_, of the monoacetyl isomers. **Table S2.** Total energies, Gibbs free energies at (298 K) and structural properties for the 1:1 AlCl_3_ complexes, 4-AcAlCl_3_,y′-Ac and 4-Ac,y′-AcAlCl_3_, of the acetyl substituted 4-acetyl-3,3′-dimethylbiphenyls. **Table S3.** Total energies, Gibbs free energies at (298 K) and structural properties for the 1:2 AlCl_3_ complexes, 4,y′-diAcAlCl_3_, of the four acetyl substituted 4-acetyl-3,3′-dimethylbiphenyls. **Scheme S2.** Illustrations of the AlCl_3_ complexed σ-complexes. **Table S4.** Total energies, *E*, Gibbs free energies at 298 K, *G*_298_, and structural properties for the AlCl_3_ complexed σ-complexes, x-AcAlCl_3_,x-H^+^. **Table S5.** Total energies, *E*, Gibbs free energies at 298 K, *G*_298_, and structural properties for the 1:1 AlCl_3_ complexed σ-complexes, 4-AcAlCl_3_,y′-Ac,y′-H^+^ and 4-Ac,y′-AcAlCl_3_,y′-H^+^. **Table S6.** Total energies, *E*, Gibbs free energies at 298 K, *G*_298_, and structural properties for the 1:2 AlCl_3_ complexed σ-complexes, 4-AcAlCl_3_,y**′**-AcAlCl_3_,y′-H^+^. Click here for file
