# Supplementary material for: A reactivity-selectivity study of the Friedel-Crafts acetylation of 3,3′-dimethylbiphenyl and the oxidation of the acetyl derivatives
Source: Chem Cent J. 2012 Jun 8;6:52. doi: 10.1186/1752-153X-6-52 (PMC3505176; doi:10.1186/1752-153X-6-52)
Supplement: Additional file 1 — Scheme S1. Illustrations of the AlCl3 complexes of the ketones. Table S1. Total energies, Gibbs free energies (298 K) and structural properties for the AlCl3 complexes, x-AcAlCl3, of the monoacetyl isomers. Table S2. Total energies, Gibbs free energies at (298 K) and structural properties for the 1:1 AlCl3 complexes, 4-AcAlCl3,y′-Ac and 4-Ac,y′-AcAlCl3, of the acetyl substituted 4-acetyl-3,3′-dimethylbiphenyls. Table S3. Total energies, Gibbs free energies at (298 K) and structural properties for the 1:2 AlCl3 complexes, 4,y′-diAcAlCl3, of the four acetyl substituted 4-acetyl-3,3′-dimethylbiphenyls. Scheme S2. Illustrations of the AlCl3 complexed σ-complexes. Table S4. Total energies, E, Gibbs free energies at 298 K, G298, and structural properties for the AlCl3 complexed σ-complexes, x-AcAlCl3,x-H+. Table S5. Total energies, E, Gibbs free energies at 298 K, G298, and structural properties for the 1:1 AlCl3 complexed σ-complexes, 4-AcAlCl3,y′-Ac,y′-H+ and 4-Ac,y′-AcAlCl3,y′-H+. Table S6. Total energies, E, Gibbs free energies at 298 K, G298, and structural properties for the 1:2 AlCl3 complexed σ-complexes, 4-AcAlCl3,y′-AcAlCl3,y′-H+. [file 1752-153X-6-52-S1.docx]

Additional file 1

**Reactivity-selectivity study of the Friedel-Crafts acetylation of 3,3'-dimethylbiphenyl and the oxidation of the acetyl derivatives**

Salam J.J. Titinchi^a*^, Fadhil S. Kamounah,^b^ Hanna S. Abbo^a^ and Ole Hammerich^c*^

*^a^Department of Chemistry, University of the Western Cape, Private Bag X17, Bellville 7535, South Africa*

*^b^CISMI, Department of Science, Systems and Models, Roskilde University, Universitetsvej 1, P.O. Box 260, DK-4000  Roskilde, Denmark*

*^c^Department of Chemistry, University of Copenhagen, Universitetsparken 5, DK-2100 Copenhagen Ø, Denmark*

The structures of the 1:1 AlCl_3_ complex of 4-Ac and the 1:1 and 1:2 complexes of 4,4′-diAc are shown in Scheme S1 as an illustration. The scheme also shows the types of abbreviations used in Tables S1-S3.

**Scheme S1:** Illustrations of the AlCl_3_ complexes of the ketones

Table S1: Total energies, Gibbs free energies (298K) and structural properties for the AlCl_3_ complexes, x-AcAlCl_3_, of the monoacetyl isomers.

| **Substituent** | **Total energy**  ***E* (a.u.)** | ***G*_298_ (a.u.)** | ***G*_298_ relative to**  **5-AcAlCl_3_**  **(kJ mol^-1^)** | **θ^a^**  **(degrees)** | **Φ^b^**  **(degrees)** |
| --- | --- | --- | --- | --- | --- |
| 2-AcAlCl_3_ | -2317.893807 | -2317.667213 | 42.3 | 48.1 | 44.0 |
| 4-AcAlCl_3_ | -2317.901782 | -2317.676534 | 17.8 | 35.0 | 11.4 |
| 5-AcAlCl_3_ | -2317.904075 | -2317.683330 | 0 | 40.0 | 0.8 |
| 6-AcAlCl_3_ | -2317.898779 | -2317.674456 | 23.3 | 49.3 | 32.1 |

Results from DFT B3LYP 6-31G(d,p) calculations. ^a^Dihedral angle between the two benzene rings taken as the average of the C2-C1-C1′-C6′ and C6-C1-C1′-C2′ dihedral angles. ^b^Dihedral angle between the carbonyl group and the benzene ring to which it is attached taken as the C_Ar_-C_Ar_-C=O dihedral angle.

Table S2: Total energies, Gibbs free energies at (298K) and structural properties for the 1:1 AlCl_3_ complexes, 4-AcAlCl_3_,y′-Ac and 4-Ac,y′-AcAlCl_3_, of the acetyl substituted 4-acetyl-3,3′-dimethylbiphenyls.

| **Substituent** | **Total energy**  ***E* (a.u.)** | ***G*_298_ (a.u.)** | ***G*_298_ relative to**  **4-Ac,5’-AcAlCl_3_**  **(kJ mol^-1^)** | **θ^a^**  **(degrees)** | **φ^b^**  **(degrees)** | |
| --- | --- | --- | --- | --- | --- | --- |
| 4-AcAlCl_3_,2′-Ac | -2470.537663 | -2470.279772 | 33.8 | 46.4 | 13.6 | 65.7 |
| 4-AcAlCl_3_,4′-Ac | -2470.546420 | -2470.287484 | 13.6 | 34.9 | 13.6 | 0.8 |
| 4-AcAlCl_3_,5′-Ac | -2470.549901 | -2470.292243 | 0.8 | 36.9 | 11.5 | 0.8 |
| 4-AcAlCl_3_,6′-Ac | -2470.540272 | -2470.283870 | 23.1 | 45.6 | 14.6 | 45.8 |
| 4-Ac,2′-AcAlCl_3_ | -2470.538015 | -2470.278460 | 37.3 | 48.0 | 1.3 | 44.8 |
| 4-Ac,4′-AcAlCl_3_ | -2470.546420 | -2470.287484 | 13.6 | 34.9 | 13.6 | 0.8 |
| 4-Ac,5′-AcAlCl_3_ | -2470.549270 | -2470.292662 | 0 | 36.6 | 1.6 | 3.5 |
| 4-Ac,6′-AcAlCl_3_ | -2470.543267 | -2470.287712 | 13.0 | 50.3 | 1.2 | 30.7 |

Results from DFT B3LYP 6-31G(d,p) calculations. ^a^Dihedral angle between the two benzene rings taken as the average of the C2-C1-C1′-C6′ and C6-C1-C1′-C2′ dihedral angles. ^b^Dihedral angle between the carbonyl group and the benzene ring to which it is attached taken as the C_Ar_-C_Ar_-C=O dihedral angle. The angle for 4-Ac is given in the column to the left; the angle for y′-Ac in the column to the right.

Table S3: Total energies, Gibbs free energies at (298K) and structural properties for the 1:2 AlCl_3_ complexes, 4,y′-diAcAlCl_3_, of the four acetyl substituted 4-acetyl-3,3′-dimethylbiphenyls.

| **Substituent** | **Total energy**  ***E* (a.u.)** | ***G*_298_ (a.u.)** | ***G*_298_ relative to**  **4,5’- diAcAlCl_3_**  **(kJ mol^-1^)** | **θ^a^**  **(degrees)** | **φ^b^**  **(degrees)** | |
| --- | --- | --- | --- | --- | --- | --- |
| 4,2′- diAcAlCl_3_ | -4093.823700 | -4093.568900 | 32.7 | 47.1 | 14.0 | 46.6 |
| 4,4′- diAcAlCl_3_ | -4093.832367 | -4093.578646 | 7.1 | 37.2 | 15.9 | 15.9 |
| 4,5′- diAcAlCl_3_ | -4093.834362 | -4093.581353 | 0 | 38.4 | 0.1 | 0.5 |
| 4,6′- diAcAlCl_3_ | -4093.828644 | -4093.576198 | 13.5 | 49.2 | 13.9 | 32.3 |

Results from DFT B3LYP 6-31G(d,p) calculations. ^a^Dihedral angle between the two benzene rings taken as the average of the C2-C1-C1′-C6′ and C6-C1-C1′-C2′ dihedral angles. ^b^Dihedral angle between the carbonyl group and the benzene ring to which it is attached taken as the C_Ar_-C_Ar_-C=O dihedral angle. The angle for 4-Ac is given in the column to the left; the angle for y′-Ac in the column to the right.

The structures of the 1:1 AlCl_3_ complex of 4-Ac,4-H^+^ and the 1:1 and 1:2 complexes of 4-Ac,4′-Ac,4′-H^+^ are shown in Scheme S2 as an illustration. The scheme also shows the types of abbreviations used in Tables S4-S6.

**Scheme S2:** Illustrations of the AlCl_3_ complexed σ-complexes.

Table S4: Total energies, *E*, Gibbs free energies at 298K, *G*_298_, and structural properties for the AlCl_3_ complexed σ-complexes, x-AcAlCl_3_,x-H^+^.

| **Substituent** | **Total energy**  ***E* (a.u.)** | ***G*_298_ (a.u.)** | ***G*_298_ relative to 4-Ac,4-H^+^**  **(kJ mol^-1^)** | **θ^a^**  **(degrees)** |
| --- | --- | --- | --- | --- |
| 2-AcAlCl_3_,2-H^+^ | -2318.201261 | -2317.964798 | 19.9 | 18.1 |
| 4-AcAlCl_3_,4-H^+^ | -2318.206324 | -2317.972382 | 0 | 20.4 |
| 5-AcAlCl_3_,5-H^+^ | -2318.177915 | -2317.947637 | 65.0 | 37.8 |
| 6-AcAlCl_3_,6-H^+^ | -2318.204007 | -2317.970706 | 4.4 | 24.8 |

Results from DFT B3LYP 6-31G(d,p) calculations. ^a^Dihedral angle between the two benzene rings taken as the average of the C2-C1-C1′-C6′ and C6-C1-C1′-C2′ dihedral angles.

Table S5. Total energies, *E*, Gibbs free energies at 298K, *G*_298_, and structural properties for the 1:1 AlCl_3_ complexed σ-complexes, 4-AcAlCl_3_,y′-Ac,y′-H^+^ and 4-Ac,y′-AcAlCl_3_,y′-H^+^.

| **Substituents** | **Total energy**  ***E* (a.u.)** | ***G*_298_ (a.u.)** | ***G*_298_ relative to**  **4-AcCl_3_,6′-Ac,6′-H^+^**  **(kJ mol^-1^)** | **θ^a^**  **(degrees)** |
| --- | --- | --- | --- | --- |
| 4-AcAlCl_3_,2’-Ac,2’-H^+^ | -2470.855080 | -2470.587889 | 6.2 | 35.7 |
| 4-AcAlCl_3_,4’-Ac,4’-H^+^ | -2470.856592 | -2470.588795 | 3.8 | 27.7 |
| 4-AcAlCl_3_,5’-Ac,5’-H^+^ | -2470.847181 | -2470.581577 | 22.7 | 38.6 |
| 4-AcAlCl_3_,6’-Ac,6’-H^+^ | -2470.857058 | -2470.590236 | 0 | 35.9 |
| 4-Ac,2’-AcAlCl_3_,2’-H^+^ | -2470.838289 | -2470.571515 | 49.1 | 25.4 |
| 4-Ac,4’-AcAlCl_3_,4’-H^+^ | -2470.845168 | -2470.578712 | 30.3 | 21.4 |
| 4-Ac,5’-AcAlCl_3_,5’-H^+^ | -2470.819168 | -2470.554413 | 94.0 | 38.5 |
| 4-Ac,6’-AcAlCl_3_,6’-H^+^ | -2470.843676 | -2470.577488 | 33.5 | 25.2 |

Results from DFT B3LYP 6-31G(d,p) calculations. ^a^Dihedral angle between the two benzene rings taken as the average of the C2-C1-C1′-C6′ and C6-C1-C1′-C2′ dihedral angles.

Table S6. Total energies, *E*, Gibbs free energies at 298K, *G*_298_, and structural properties for the 1:2 AlCl_3_ complexed σ-complexes, 4-AcAlCl_3_,y**′**-AcAlCl_3_,y′-H^+^.

| **Substituents** | **Total energy**  ***E* (a.u.)** | ***G*_298_ (a.u.)** | ***G*_298_ relative to**  **4-AcAlCl_3_,4′-AcAlCl_3_,4′-H^+^**  **(kJ mol^-1^)** | **θ^a^**  **(degrees)** |
| --- | --- | --- | --- | --- |
| 4-AcAlCl_3_,2′-AcAlCl_3_,2′-H^+^ | -4094.106574 | -4093.846493 | 12.3 | 33.1 |
| 4-AcAlCl_3_,4′-AcAlCl_3_,4′-H^+^ | -4094.112921 | -4093.851195 | 0 | 25.2 |
| 4-AcAlCl_3_,5′-AcAlCl_3_,5′-H^+^ | -4094.091448 | -4093.830797 | 53.5 | 41.6 |
| 4-AcAlCl_3_,6′-AcAlCl_3_,6′-H^+^ | -4094.112655 | -4093.850903 | 0.8 | 29.8 |

Results from DFT B3LYP 6-31G(d,p) calculations. ^a^Dihedral angle between the two benzene rings taken as the average of the C2-C1-C1′-C6′ and C6-C1-C1′-C2′ dihedral angles.
